# A Case Report of Horner’s Syndrome in Labour Neuroaxial Analgesia: Diagnosis, Management and Follow-Up

**DOI:** 10.3390/reports9030289

**Published:** 2026-08-28

**Authors:** Yong-Shun Thoo, Mariana Fernandes, Marie-Joe Dib, Corinne Grandjean, Ouanes Amine Ben Saad

**Affiliations:** 1 Emergency Department, Vaud University Hospital (CHUV), 1005 Lausanne, Switzerland; 2 Service of Anaesthesiology and Reanimation, Geneva University Hospital (HUG), 1205 Geneva, Switzerland; 3 Service of Anaesthesiology and Reanimation, Fribourg Hospital, 1708 Fribourg, Switzerland

**Keywords:** Horner’s syndrome, obstetric analgesia, lumbar epidural, obstetric anaesthesia, analgesia for labour

## Abstract

**Background and Clinical Significance**: Horner’s syndrome is an uncommon but recognised complication of neuroaxial analgesia in obstetric procedures, with an incidence of 0.4–4%. **Case Presentation:** This case report describes a 28-year-old primigravida who developed unilateral Horner’s syndrome approximately one hour after lumbar epidural analgesia placement for labour pain relief. Upon examination the patient presented with left-sided ptosis, anisocoria, and unexpected sensory blockade extending to the T3–T4 level, accompanied by localised numbness in the left breast and transient upper limb paraesthesia. The pathophysiology of Horner’s syndrome involves cephalad spread of local anaesthetic toward the superior cervical sympathetic chain, disrupting sympathetic innervation to the ocular and facial areas. Primary and secondary damage to the sympathetic pathways in the central nervous offer a wide differential diagnosis. The management was conservative, including reduction in epidural bolus infusion rates and careful monitoring. The patient experienced complete resolution of all symptoms within five hours and delivered vaginally without complications. **Conclusions:** This case demonstrates the characteristically benign and self-limited nature of this complication, emphasising the importance of clinical awareness, proper reassurance of patients, and avoidance of unnecessary diagnostic testing.

## 1. Introduction and Clinical Significance

Horner’s syndrome is a recognised complication of neuraxial analgesia in obstetrics with an incidence between 0.4 and 4% [1,2]. It has been suggested that it occurs at a higher frequency compared to non-obstetric populations receiving lumbar epidural techniques [2,3], though its true incidence in the non-obstetric population is unknown. Though the clinical course is usually benign, here we present a clinical case where we considered alternative diagnoses for neurological deficits after lumbar epidural analgesia and when clinical work up should be considered.

## 2. Case Presentation

A 28-year-old primigravida (G1P0) patient, with a height of 165 cm and a weight of 74 kg (BMI = 27 kg/m^2^), was admitted to our maternity ward at 38 weeks of gestation in spontaneous labour. She had no significant past medical history. Review of medical records revealed that she received low-dose daily aspirin prophylaxis (100 mg/d) until 36 weeks of gestation for an “increased thromboembolic risk”. No thromboembolic risk scores were registered in the medical records. The patient had an uncomplicated pregnancy until admission for labour. Upon admission, the patient presented with regular uterine contractions and a cervical dilation of 1 cm. At that point, she requested epidural analgesia. In our institution, informed consent for the procedure is obtained in advance. Women receive information sheets, access to an educational video and informed consent forms about epidural analgesia during routine obstetrical check-ups.

The patient had an epidural catheter placed in a sitting position, using a midline L3–L4 interspace approach. A single attempt was sufficient to advance a standard 18 G Tuohy needle using a one-handed needle advancement technique with continuous pressure. Loss of resistance to saline was identified at 4.5 cm. A multi-orifice epidural catheter was then inserted to a depth of 10 cm at the skin, leaving 5.5 cm of catheter within the epidural space. After correct placement confirmation with three negative aspiration tests, a test dose of 3 mL of bupivacaine 0.125% combined with fentanyl at 2 μg/mL was administered. Our patient had no motor symptoms after the test dose that could indicate placement of the catheter in the intrathecal space. The anaethetist added 7 mL of the solution to complete the dose. Our institutional protocol for maintenance of analgesia includes programmed intermittent epidural boluses (PIEB) of bupivacaine 0.125% with fentanyl at 2 μg/mL, delivering 5 mL/h. Furthermore, patient-controlled epidural analgesia (PCEA) is enabled, allowing self-administered boluses of 5 mL every 20 min as needed.

**Figure 1:**
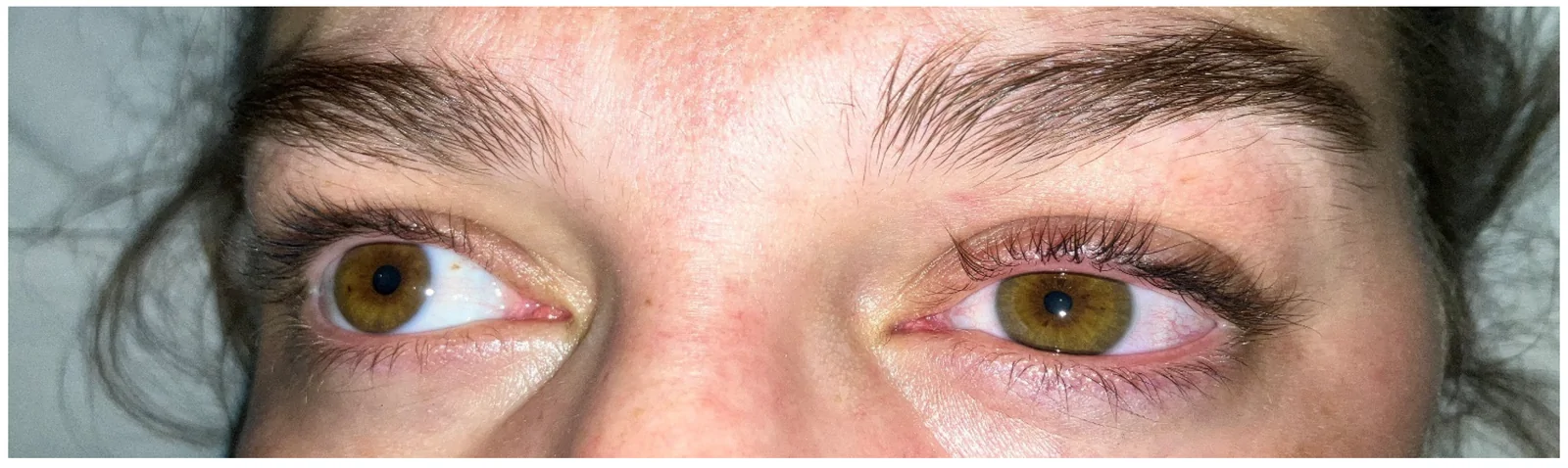
Left-sided miosis and ptosis.

Approximately 2 h later, the midwife requested an evaluation by the anaesthesia team. The patient had reported the onset of left-sided ptosis, accompanied by numbness in the left breast, localised just below the clavicle. She also experienced transient numbness in the left arm and described reduced sensation to the saline infusion from a peripheral intravenous catheter placed on the posterolateral aspect of the left forearm. The symptoms had begun approximately one hour after epidural catheter placement and were regressing by the time of evaluation. The patient was otherwise comfortable and stable. On clinical examination, we noted left-sided miosis, ptosis and conjunctival hyperemia (see Figure 1). Cold sensation testing revealed a sensory block level at T4 on the right and T3 on the left. There were no objective sensory or motor deficits in the arms or legs. We considered multiple differential diagnoses, but suspected epidural analgesia involvement. Since symptoms had regressed at the time of assessment, we adopted a “wait and see” approach. We directly informed our patient and her partner that the complication was probably related to the epidural anaesthesia. The hourly programmed intermittent boluses were reduced to 3 mL/h, and the patient was advised to notify care providers before using any patient-controlled boluses. No further boluses were required for analgesia. 

The neurological impairments did not affect delivery or the newborn. Spontaneous vaginal delivery occurred five hours after epidural placement, without obstetric or new-born complications. The patient reported complete resolution of her symptoms within five hours. Our anaesthesia team completed daily follow-up for the next 48 h and we reported no neurological-related symptoms. No additional adverse events or necessary medical tests occurred during follow-up. The parturient was discharged from the maternity ward 72 h after delivery.

## 3. Discussion

Acute neurological deficits during pregnancy or delivery warrant immediate evaluation. In our case, our patient exhibited typical signs for incomplete Horner’s syndrome (myosis and ptosis) and sensory symptoms of the left arm. Her personal medical records were negative for neurological, cardiovascular or oncologic illnesses. The records did not fully explain the reasoning for low-dose prophylactic aspirin, and we suspect it was for pre-eclampsia prophylaxis. Aspirin had been stopped two weeks prior to admission. Horner’s syndrome or oculosympathetic paresis occurs when a lesion affects the sympathetic pathways of the central nervous system. The typical triad of clinical signs are miosis (leading to anisocoria), ptosis and facial anhidrosis. The pathway (first-order neurons) starts from the hypothalamus and descends the ipsilateral brainstem until reaching the spinal cord between C8-T2. Second-order neurons exit the spinal cord, pass over the lung apex, and synapse in the superior cervical ganglion (C3-C4). Third-order postganglionic neurons follow the internal and external carotid arteries. The branches following the external carotid arteries innervate blood vessels and sweat glands of the face. The branches following the internal carotid arteries pass through the cavernous sinus and divide into two branches. One branch reaches the eyelid muscles and lacrimal glands, and the second joins the ophthalmic division of the trigeminal nerve and enters the orbit, innervating the iris dilator muscle [4,5]. Horner’s syndrome has a wide differential diagnosis that include primary diseases of the central nervous system or secondary diseases that may compress or affect the sympathetic pathway in the head and neck [5]. Head/neck pain, oculomotor deficits, arm and/or leg weakness, diplopia and vertigo associated with acute Horner’s syndrome are red flags that should prompt consideration for further investigations. Stroke, carotid artery or venous thrombosis are emergencies that should be ruled out. Maternal stroke risk is three-fold-increased in pregnancy and the postpartum period (up to twelve weeks after delivery) [6]. Ante-, peri- and postpartum stroke should be considered as stroke may affect 30/100,000 pregnancies [7]. Traditional risks factors and specific pregnancy-related factors (hypercoagulable state of pregnancy, hypertensive disorders of pregnancy) are important mechanisms for the increased risk in pregnant or postpartum patients. Carotid or vertebral artery dissection has been associated in postpartum stroke but is rarer in the ante- or perinatal period [8] (Table 1). Rapid identification and treatment of causes of stroke are the mainstay of treatment to avoid morbidity.

Our parturient displayed no high-risk features such as head/neck pain or extensive neurological deficits. Her personal and obstetric medical records showed no evidence for alternative undiagnosed causes for Horner’s syndromes. We speculate that the prescribed aspirin may have been primary prophylaxis for pre-eclampsia [9], as to our knowledge, thromboembolic illness risk is treated with anticoagulation [10,11] rather than antiplatelet therapy. However, these therapies and associated disorders of therapy are not associated with Horner’s syndrome. Since symptom onset followed epidural analgesia, we assumed this was the most probable cause, as has been described in the literature. Other causes for Horner’s syndrome may be known in the patient’s medical history before entering the delivery ward. Epidural anaesthesia and other locoregional anaesthetic approaches can cause acute Horner’s syndrome. In our case, no red flags were identified on clinical evaluation or examination of medical records. Since symptom onset followed epidural analgesia, we assumed this was the most probable cause. We chose to monitor symptoms and did not order additional exams since the clinical course had been favourable and did not include red flag features. It has been reported that Horner’s syndrome occurs more frequently in pregnant patients, although the true incidence is unclear. The fundamental pathophysiology in pregnant patients involves diffusion of the local anaesthetics from the lumbar segments towards the superior cervical sympathetic chain [12]. This significant cephalic spread interferes with the pathways innervating the face and eyes, causing sympathetic nerve disruption to the ocular and facial areas. Pregnancy-related anatomical changes in the epidural space (narrowing), mechanical effects of contractions that can increase epidural pressure, hormonal changes on neuronal sensitivity to local anaesthetics and improper placement of the catheter in the subdural space have been proposed as pathophysiological mechanisms facilitating the occurrence of Horner’s syndrome during labour. Our case presentation illustrates several characteristic features of Horner’s syndrome occurring during labour epidural analgesia [13]. The onset of symptoms is typically unilateral and occurs within one hour after epidural injection. Associated findings may include not only the classic triad but also other neurological manifestations such as unilateral upper limb paraesthesia [14] and high sensory blockade levels [15,16] extending to thoracic or even cervical dermatomes. The distribution of the sensory symptoms often have an unexpected pattern of distribution above the expected level, occasionally spreading from sacral to thoracic or cervical dermatomes. The development of this unexpected high sensory blockade, in association with ptosis, miosis, and facial anhidrosis, should prompt the clinician to consider the diagnosis of an iatrogenic Horner’s syndrome rather than a primary neurologic condition. In some cases, patients may experience transient autonomic symptoms including brief episodes of hypotension and bradycardia secondary to sympathetic dysfunction. 

The clinical work-up involves taking vitals to ensure haemodynamic-respiratory stability and performing thorough neurological examination searching for typical signs of Horner’s syndrome and associated neurological signs. Unusual symptoms, such as cranial nerve palsies, pain (head and neck), motor symptoms, extensive sensory symptoms, persistent/non-regressive symptoms, or persistent haemodynamic instability should alert the physician to consider an alternative diagnosis or an extensive block. To effectively manage patients presenting with Horner’s Syndrome, healthcare providers (anaesthetists, obstetricians and midwives) must be aware of this uncommon problem and distinguish it from other more serious conditions. The side effects of Horner’s syndrome due to epidural analgesia are typically benign and self-limited, and are thus reassuring characteristics of Horner’s syndrome. Ocular symptom resolution typically occurs within hours of symptom onset; published cases document resolution periods ranging from 2.5 to 4 h after onset [13]. The vast majority of reported cases demonstrate complete spontaneous resolution without lasting neurological consequences. This transient course reflects the temporary nature of local anaesthetic effects and the gradual normalisation of sympathetic function as medications are metabolised and reabsorbed. The absence of permanent neurological damage and favourable maternal and neonatal outcomes in nearly all documented cases supports conservative expectant management in uncomplicated presentations [13]. The presence of this complication does not ordinarily contraindicate successful labour analgesia continuation or vaginal delivery. Haemodynamic monitoring deserves particular attention, as some patients develop brief periods of hypotension due to sympathetic dysfunction [17]. While these haemodynamic changes are typically modest and self-resolving, continuous monitoring of maternal vital signs and foetal heart rate (cardiotocography) remains essential throughout the labour process and delivery. Fluid boluses and vasopressors may be required to maintain blood pressure. Horner’s syndrome during neuraxial analgesia has occurred using a wide variety of different local anaesthetics and administration methods (bolus or continuous infusions) [18]. Local anaesthetic management strategies after the diagnosis may include adjusting epidural infusion rates, continuing labour analgesia with epidural boluses or providing alternative oral or intravenous analgesia. In this situation, the on-call anaesthetic team chose to reduce the dosage of the programmed intermittent hourly boluses without the patient noticing a recurrence of symptoms. In some reported cases, misplacement of the epidural catheter is described to explain the onset of Horner’s syndrome. Subdural block has been associated with extensive sensory deficits disproportionate to local anaesthetic dosage, cranial nerve involvement, and inadequate neuraxial pain management [19,20]. Spread of local anaesthetics from the subdural to the intracranial space can lead to life threatening cardio-respiratory and neurological compromise [3]. Suspected dural puncture may therefore warrant disuse and removal of the catheter. In order to understand the localisation of epidural catheters (epidural, subdural or subarachnoid space) [21,22], epidurograms (using a contrast agent and plain radiography), computed tomography and magnetic resonance angiography have previously been performed in obstetric patients with Horner’s syndrome after labour. However, these exams will be difficult to perform during labour and have no implication in routine clinical management. It may therefore be difficult to distinguish between a subdural and epidural block with moderate symptoms. When epidural catheters must be replaced or repositioned due to inadequate pain management or a suspected subdural block in patients, recurrent symptoms may occur with subsequent dosing [23] and even in subsequent pregnancies [19]. This possibility warrants careful consideration when managing patients with a history of this complication [24,25]. In our case, it is unknown whether the syndrome was triggered by the subsequent dosing from the hourly epidural catheter bolus since onset of symptoms occurred approximately an hour after the initial procedure. We informed the parturient that there is no formal contraindication to re-attempt neuraxial anaesthetic approaches in further pregnancies but should be reported in future preoperative consultations. Follow-up to ensure complete regression and resolution of symptoms in the hours after the procedure is necessary to confirm the diagnosis for Horner’s syndrome due to epidural analgesia during the hospital stay. Reassurance should be offered to patients for future pregnancies. If symptoms had persisted after delivery, a full neurological consultation and urgent medical imaging would have been required. 

## 4. Conclusions

Unilateral Horner’s syndrome associated with lumbar epidural anaesthesia for delivery is an uncommon complication of obstetric anaesthesia. However, there have been more reported cases in recent years. The symptoms in this population are usually benign without any long-term complications, but they may still create anxiety for the patient and the clinical team. The educational implications of this complication extend to anaesthesiologists and obstetric care teams. Understanding the pathophysiology, but also the alternative diagnoses associated with this syndrome, should help all members of the perinatal care team to understand when further intervention is necessary to avoid unnecessary diagnostic or therapeutic interventions. In most situations, conservative management and follow-up are the mainstay of treatment. 

## Figures and Tables

**Table 1:** Differential diagnosis for Horner’s syndrome in the pregnant patient, according to localisation along the sympathetic pathway.

Central (Hypothalamus to Spinal Cord T2)	Preganglionic Neurons (Spinal Cord to Sympathetic Ganglionic Chain)	Postganglionic Neurons
Hypothalamus Stroke Cancer Demyelinating diseases Brainstem Stroke (including Wallenberg syndrome) Arterial venous malformations Syringomelia Cancer Spinal cord lesions Trauma Arterial venous malformations or infarction Demyelinating diseases	Head and neck cancers Chest cancers Lung cancer (pancoast) Lymphoma Breast cancer Aortic, subclavian or main carotid artery dissection Iatrogenic Epidural/spinal anaesthesia or analgesia Heart and chest surgery (affecting the passage of nerves along the lung apex)	Internal/external carotid artery Thrombosis Dissection Cavernous sinus thrombosis

## Data Availability

All relevant data are within the manuscript.

## Abbreviations

The following abbreviations are used in this manuscript:

BMI	Body Mass Index
PIEB	Programmed Intermittent Epidural Boluses
PCEA	Patient-Controlled Epidural Analgesia
